# The prelimbic cortex is critical for context-dependent fear expression

**DOI:** 10.3389/fnbeh.2013.00073

**Published:** 2013-06-21

**Authors:** Eun Joo Kim, Namsoo Kim, Hyun Taek Kim, June-Seek Choi

**Affiliations:** Department of Psychology, Korea UniversitySeoul, Republic of Korea

**Keywords:** prelimbic cortex, fear discrimination, context, amygdala, hippocampus

## Abstract

The ability to regulate emotional responses in various circumstances would provide adaptive advantages for an individual. Using a context-dependent fear discrimination (CDFD) task in which the tone conditioned stimulus (CS) is paired with the footshock unconditioned stimulus (US) in one context but presented alone in another context, we investigated the role of the prelimbic (PL) cortex in contextual modulation of the conditioned fear response. After 3 days of CDFD training, rats froze more to the CS presented in the fearful than in the safe context. Following bilateral lesions of the PL, rats showed similar levels of freezing to the CS in both contexts, in contrast to the sham-lesioned control animals. The lesions did not impair the rats' ability to discriminate contexts *per se*, as indicated by intact differential responses in a separate experiment which employed a simple context discrimination task. Consistent with the lesion data, single-unit recordings from the PL showed that the majority of CS-responsive neurons fired at a higher rate in the fearful context than in the safe context, paralleling the behavioral discrimination. Taken together, the current results suggest that the PL is involved in selective expression of conditioned fear to an explicit (tone) cue that is fully dependent on contextual information.

## Introduction

Learning to express context-appropriate fear responses constitutes an important survival strategy since a cue can signal danger in one context but not necessarily in another. Most forms of defense reaction impose heavy costs on the animal, consuming resources and sacrificing opportunities to perform other behaviors (Choi and Kim, 2010). Therefore, it is beneficial for an animal to selectively express fear when contextual cues provide unambiguous information.

Among the brain structures implicated in contextual processing, the hippocampus has been shown to mediate context-specific expressions of fear responses (Corcoran and Maren, 2001; Maren and Hobin, 2007). In addition, recent human imaging studies indicate that the medial prefrontal cortex (mPFC) and its interaction with the hippocampus might play a key role in regulating the fear response (Kalisch et al., 2006; Milad et al., 2007). To date, however, little is known about the role of the mPFC in context-dependent modulation of fear expression at the neurophysiological level.

An emerging body of evidence suggests that the mPFC participates in contextual processing and fear expression. For instance, neurons in the mPFC display goal-directed firing pattern indicating that they encode relevant spatial information, a crucial component of context (Hok et al., 2005). In addition, electrolytic lesions or pharmacological inactivation of the mPFC impaired contextual control of the instrumental response in a conflict situation (Haddon and Killcross, 2006; Marquis et al., 2007). The mPFC is also involved in fear expression as inactivation and microstimulation of the mPFC have been shown to alter fear expression levels (Sierra-Mercado et al., 2006; Vidal-Gonzalez et al., 2006; Corcoran and Quirk, 2007). Single-unit activities in the mPFC have been found to correlate with the magnitude of conditioned freezing behavior (Gilmartin and McEchron, 2005; Burgos-Robles et al., 2009).

The prelimbic (PL) area, a subregion of the mPFC, receives a number of afferent projections from the hippocampus and multiple sensory cortical areas and sends efferent projections to the amygdala (Jay and Witter, 1991; Hoover and Vertes, 2007). Since both the hippocampus (Kim and Fanselow, 1992; Phillips and LeDoux, 1992) and the amygdala (Kim and Davis, 1993; LeDoux, 2000) have been shown to play distinctive roles in fear memory encoding and expression, the PL might be one of the components that integrates sensory and/or contextual information to produce context-appropriate fear responses.

To examine the role of the PL in the contextual regulation of fear response, a context-dependent fear discrimination (CDFD) task, modified from a discrimination task previously used to demonstrate a contextual control of fear response in rats (Bouton and Swartzentruber, 1986), was employed. Two contexts were used: one in which the CS and the US were paired, and the other in which the CS was presented without the US. Since the same CS signaled contrasting events, danger or safety, context serves as the disambiguating cue. We tested whether and how the PL is involved in CDFD with a series of lesion and single-unit recording experiments.

## Materials and methods

### Subjects

Male Sprague Dawley rats (initially weighing 215–245 g; Orient Bio, Kyunggi-do, Korea) were housed individually on a 12-h reversed light/dark cycle (lights on at 9:00 P.M.) with *ad libitum* access to food and water. All experimental procedures were conducted during the dark phase of the cycle and in compliance with the National Institutes of Health (NIH) guidelines.

### Electrolytic lesion

Rats were fully anesthetized using sodium pentobarbital (60 mg/kg, i.p.) and mounted on a stereotaxic apparatus (David Kopf Instruments, Tujunga, CA). The scalp was incised, and small holes were drilled for electrode insertion. For electrolytic lesions, stainless steel electrodes (0.3 mm in diameter) insulated with epoxy, except for 0.5 mm at the tip, were lowered bilaterally into the PL (2 sites for each hemisphere: 2.5 mm anterior, 0.6 mm lateral, 4.0 mm ventral; and 3.5 mm anterior, 0.6 mm lateral, 3.8 mm ventral to bregma). The lesion was made by passing anodal current (1.0 mA for 15 s) at each site. Sham animals were treated in the same manner except that no current was passed.

### Recording electrode implantation

Under anesthesia, one or two bundles of eight fine wires (25 μm, formvar-insulated NiCr, A-M systems Inc., Everett, WA) aimed at the PL (3.2 mm anterior, ±0.6 mm lateral to bregma, and 2.8–3.2 mm ventral to the brain surface), were lowered uni- or bilaterally. The tips of the electrodes were cut and gold-plated to yield impedance of 200–500 kΩ at 1 kHz. The electrode assembly was secured to the skull with six anchoring screws and dental cement. Four of the skull screws were used as the ground. Following surgery, rats were allowed to recover for at least 6 days before the experiment started.

### Apparatus

#### CDFD and simple context discrimination task

**Figure 1:**
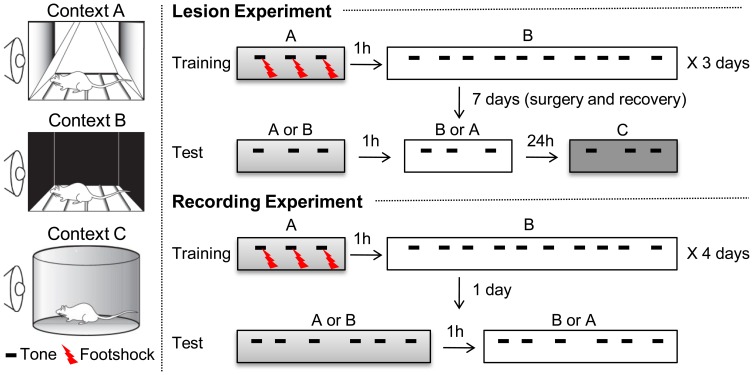
**Experimental procedure for context-dependent fear discrimination (CDFD)**. Two different contexts, denoted as A and B, were used for training. During training, rats were given two alternating sessions on the same day, separated by 1 h: one in Context A and the other in Context B. Training lasted for 3 days. In Context A, three parings of the CS and US were presented. In Context B, they were given ten CS-only trials. Training lasted for 3 days. Twenty-four hours after the final training session, the PL lesion and SHAM group received surgery for electrolytic lesion or sham lesion, respectively. After 7 days of recovery, the rats were given two separate test sessions in Context A and B. An additional testing session, in a new context (Context C), was given to determine the magnitude of the CR unaffected by the conditioning contexts. For the recording experiment, the same training procedure was used for 4 days and the recording session was given only 24 h after the last training session. Testing in Context C was omitted.

For the CDFD test, two contexts (Context A and B) with distinctively different configurations, odor, and color were used for training and testing. In addition, a novel context (Context C) was used for additional testing. Context A (fearful) and B (safe) consisted of one of the following context combinations (counterbalanced across animals): (i) a black Plexiglas box (30 × 25 × 25 cm) and citrus odor under blue illumination or (ii) a transparent Plexiglas box (27 × 25 × 34 cm) and mint odor under red illumination with an inverted V-shaped foam board inserted below the ceiling. Context C consisted of a white Plexiglas cylinder (25 cm in diameter) with cinnamon odor under white illumination (see Figure 1). In Context A and B, a grid floor, composed of 16 stainless steel rods (0.5 cm in diameter, 1.5 cm apart), was connected to a scrambled shock generator (Coulbourn Instruments, Whitehall, PA). A small speaker (8 × 4 cm, 8 Ω), located on the side wall, and connected to an audio amplifier and a function generator, was used to supply the tone CS. The CS and US were controlled by a custom-written program (Smarteye, Smartech, Madison, WI). Each chamber was placed in a sound attenuation cubicle (48 × 55 × 45 cm). A video camera, mounted on the ceiling of the cubicle, monitored the animals' behavior. All sessions were recorded on a video recorder for offline analysis.

#### Open field test

A square arena (77 × 77 cm, wall height: 25 cm), placed in a room surrounded by a black curtain and illuminated with indirect halogen lighting, was used for the open field test. The center area was defined as a square section in the center (46.2 × 46.2 cm). A video camera and a webcam monitored and recorded the rats' spontaneous activity. The movement trajectories were recorded and analyzed with an automated tracking system (Smartrack, Smartech, Madison, WI).

#### Prepulse inhibition (PPI) test

For the PPI test, a rat was confined in a Plexiglas cylinder (10 cm in diameter, 20 cm in length) and placed on a platform supported by a load cell (CB1-K002, Dacell Co., Chungcheongbuk-Do, Korea). A small speaker (9 cm in diameter, 30 W, 8 Ω) delivered the prepulse stimulus (sine-wave tone, 4 kHz, 80 dB). The prepulse was followed by a startle stimulus (white noise, 120 dB) delivered through a tweeter (11 cm in diameter, 60 W, 8 Ω). Vertical displacement of the load cell caused by the animal's startle reaction was amplified (×1000) and digitized by a DIO card (PCI-6154, NI, Austin, TX). Stimulus presentation and data acquisition were automatically controlled by a custom program written in LabVIEW (NI). The test was performed inside a sound attenuating chamber (50 × 50 × 50 cm) to attenuate outside noise.

### Behavioral procedures

#### CDFD

As shown in Figure 1, the experimental procedure was divided into habituation, training, and testing stages. One day before training, all the animals were acclimated to Context A for 10 min and then returned to their home cages. One hour later, they were exposed to Context B for 10 min. For the discrimination training, rats were placed in Context A for 180 s before receiving three pairings of a co-terminating tone CS (30 s, 4 kHz, 75 dB) and a footshock US (0.5 s, 0.5 mA). Three minutes after the last trial, they were returned to their home cages. One hour later, they received 10 CS-only trials in Context B. These two training sessions were repeated for 3 days. The average inter-trial interval for all sessions was 180 s, ranging from 160 to 200 s. For the recording experiment, rats were trained for 4 days, rather than for 3 days. The additional training session was applied to ensure a robust contextual control of fear expression. Twenty-four hours after the last training session, half of the animals were given PL lesions (PL lesion group, *n* = 8) and the other half received sham lesions (SHAM group, *n* = 8). After 7 days of recovery, testing was conducted in Context A and then in Context B, separated by 1 h. Three CS-only trials were presented to the rats in each testing session. The order of exposure to the context was counterbalanced across animals: some rats were tested in Contest A then in B, while others were tested in B then in A. On the following day, all animals were tested in novel Context C, to which they had not been previously exposed. Three minutes after they were placed in Context C, they were given 3 CS-only trials. Freezing was analyzed during the first CS presentation period in each context. For the recording experiment, they were given 6 CS-only trials in each testing session.

#### Simple context discrimination task

All procedures were identical to those used in CDFD, except that no discrete CS was provided throughout the task. After habituation and training sessions, rats also received a PL (*n* = 8) or sham lesion (*n* = 8), followed by 7 days of recovery. They were tested in Context A and then B, or vice versa, separated by 1 h. Each test session lasted for 13 min per context. Freezing during the first 3 min was included for the analysis.

#### Open field test

Rats were placed in the center of the open field arena and their activities were recorded for 10 min. The movement trajectories were recorded and analyzed. The percentage of time spent in the central and marginal areas and the number of rearing were also measured.

#### PPI test

Four trial types were used for the PPI test: a prepulse-alone trial, a startle stimulus-alone trial, a prepulse-startle stimulus trial (i.e., the prepulse was followed by the startle stimulus after a 100-ms interval), and a no-stimulus trial. A total of 60 trials were randomly presented, separated by random intervals (5–25 s). The PPI was calculated as follows: PPI (%) = 100 × (SS-PS)/SS, in which SS denotes the average startle amplitude to the startle stimulus, and PS denotes the average startle amplitude to the prepulse–startle stimulus.

### Unit recording and data analysis

The single-unit activity was amplified by a head-stage amplifier (unity gain) and a main amplifier (×10,000), filtered between 600 and 6000 Hz, and sampled at 32 kHz using a Cheetah data acquisition system (Neuralynx, Tucson, AZ). The digitized activity was stored in a personal computer for offline analyses.

Freezing, defined as the absence of movement except for respiration (Blanchard and Blanchard, 1969), was used as the index of fear CR. Freezing was quantified by a video analysis with two experimenters who were blind to the condition of the subjects using digital stopwatches. Units were identified and classified automatically using the KlustaKwik method (written by K. D. Harris, Rutgers University, Newark, NJ) in which the energy of a spike waveform, the amplitude of the peak and valley, the first and second principal components, and the Fourier transform were used as parameters for unit isolation. Further correction was conducted using the MClust 3.3 spike sorting program (written by A. D. Redish, University of Minnesota, Minneapolis, MN). Autocorrelogram, cross-correlogram, and interspike interval histograms were constructed to verify that the isolated units did not overlap. Using Neuroexplorer (Nex Technologies, Lexington, MA), peristimulus time histograms (PSTH) were generated. The numerical results were analyzed using Matlab 6.5 or SPSS 12.01. The mean spontaneous firing rates of the units were computed from the pre-tone exposure period (180 s). Unit data were binned into 50 ms and normalized to the pre-CS baseline period (1 s). A unit was considered to be short-latency CS-responsive if the firing rate in at least one of the first 3 post-CS bins (within 150-ms window) was over 3 standard deviations (SDs) above the baseline. The unit data were also binned into 1 s and normalized to the pre-CS baseline period (20 s). A unit was considered showing sustained activity or a “persistently firing unit” (PFU) if the firing rate in two or more bins during the first 10 bins (10 s) were over 3 SDs above the baseline. For the population-level analysis, *Z-scores* were calculated for each unit and averaged. A cell-by-cell analysis was performed by computing *t*-values based on paired comparisons between the matching bins. There were thirty 1-s bins for the duration of the tone CS, and the *t*-values were calculated by summing the *Z*-score differences (Context A vs. B) for each bin.

Repeated measures analysis of variance (RM ANOVA) compared the freezing levels and unit responses to the CS in Context A to those in Context B (and Context C). *Post-hoc* tests were performed by Fisher's least significant difference when necessary. For the open field and prepulse inhibition tests, Student's *t*-test was conducted.

### Histology

Following the termination of the behavioral testing and/or recording, the rats were overdosed with pentobarbital sodium (120 mg/kg, i.p.). The electrode placements were marked by passing anodal current (10 μA for 10 s) through the tip of 2 or 3 electrodes in each bundle. Animals were then perfused with saline (0.9%) and paraformaldehyde (10%). The brain was removed, post-fixed overnight, and transferred to a 30% sucrose solution for 2–3 days. The tissue was sectioned transversely at a thickness of 50 μm. All sections were stained using cresyl violet following a reaction in a 2% potassium ferrocyanide solution to visualize iron deposits created by the lesion. The stained brain sections were analyzed using NIH Image (V.1.38) in order to quantify the extent of the lesions. The size of the lesion was calculated by dividing the lesioned area by the total area of the region of interest [PL, infralimbic area (IL), or cingulate cortex (Cg1)] identified from the atlas devised by Paxinos and Watson (1998). For the lesion experiment, only animals with substantial bilateral lesions in the PL (at least 70%) were included. For the recording experiment, only those with electrodes in the PL were included.

## Results

### The PL is necessary for CDFD

**Figure 2:**
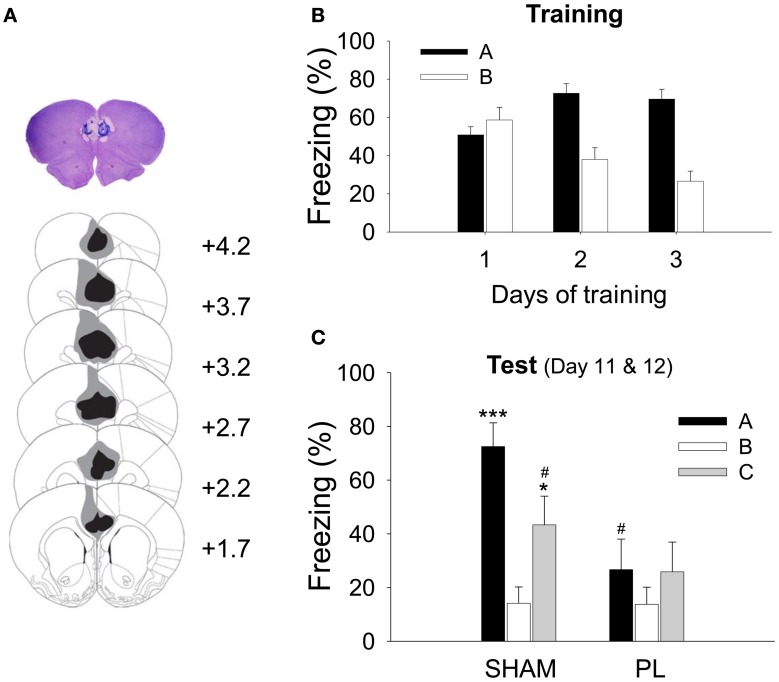
**Histological verification of the PL lesion and acquisition of CDFD. (A)** High-resolution scan of a cresyl-violet-stained coronal section shows a representative PL lesion (top), and the reconstruction shows the extent of the electrolytic lesion (bottom). The gray shading depicts the largest and the black depicts the smallest lesion on the matching coronal sections from an atlas (Paxinos and Watson, 1998). **(B)** During training, all animals gradually acquired differential responses to the CS in Context A and B. **(C)** After surgery, the SHAM group showed significantly more freezing to the CS in Context A or C than in Context B. However, the PL lesion group showed a similar level of freezing in all three contexts. ^*^ and ^***^denote *p* < 0.05 and *p* < 0.001, respectively, compared to the SHAM group in Context B; ^#^denotes *p* < 0.05 compared to the SHAM group in Context A.

As shown in Figure 2A, the lesion included most of the PL and some of the neighboring areas. The image analysis showed that the lesions destroyed most of the PL (79.9 ± 4.57%, mean ± SD). In addition, the anterior portions of the Cg1 (29.65 ± 15.72%) and the dorsal IL region (14.83 ± 16.37%) were partially damaged.

For the CDFD, the rats received 3 CS–US pairings in the fear context and 10 CS-only trials in the safe context. This discriminatory training was repeated for 3 days. Because a RM ANOVA revealed that there was no difference between groups in their freezing during training [group effect, *F*_(1, 14)_ = 0.005, *p* > 0.9; day × group interaction, *F*_2, 28_ = 0.251, *p* > 0.7; context × group interaction, *F*_1, 14_ = 0.057, *p* > 0.8; day × context × group interaction, *F*_2, 28_ = 0.190, *p* > 0.8], the two groups (SHAM and PL lesion) were collapsed for the training data. All rats showed differential response following 3 days of training (Figure 2B), as indicated by a main effect of context [context, *F*_1, 15_ = 42.948; *p* < 0.0001]. The discrimination gradually developed over the training sessions as indicated by a significant main effect of training days [day, *F*_2, 30_ = 3.567, *p* < 0.05] and interaction [day × context, *F*_2, 30_ = 20.218, *p* < 0.0001]. A *post-hoc* test showed that the differential response was evident on training days 2 (*p* < 0.0001) and 3 (*p* < 0.0001). Seven days after surgery, the rats were tested in Context A, and then in B (or B then A; counterbalanced) on the same day. On the following day, the rats were tested in Context C to determine whether the fear CR re-appears to the CS in a novel context. Figure 2C illustrates the results from these test sessions. There was a significant context effect [*F*_2, 28_ = 8.661, *p* < 0.001], showing that the rats froze more in Context A and C than in Context B. In addition, there was a significant group effect [*F*_1, 14_ = 5.995, *p* < 0.05] as well as a context × group interaction [*F*_2, 28_ = 3.564, *p* < 0.05], indicating that the SHAM animals froze more to the CS than PL lesion animals in Context A (*p* < 0.01), as confirmed by the *post-hoc* test. Further *post-hoc* tests revealed that the SHAM rats froze more to the CS in Contexts A and C than in B (*p* < 0.001 and *p* < 0.05, respectively) and more in Context A than in C (*p* < 0.05). In contrast, PL lesion rats maintained a similar level of freezing throughout the three test sessions (26.7 ± 10.18, Context A; 13.8 ± 6.22, Context B; 25.9 ± 10.88, Context C). These results indicate that the post-training PL lesion disrupted the CDFD and renewal of the fear response to the CS in a novel context.

Since the focus of the current study is the tone-evoked response, it needs to be determined whether the level of freezing discriminated the discrete tone CS from the background context. Therefore, based on the data from the SHAM group, we tested whether freezing to the tone CS in Context A (SHAM black bar as shown in Figure 2C) was greater than freezing to the context during the pre-tone baseline period (SHAM black bar as shown in Figure A2B). There was a marginally significant difference between the levels of freezing to the tone CS and that to the context [paired *t*-test, *t*_(7)_ = 1.883, *p* = 0.102]. The difference did not reach significance because some of the SHAM rats exhibited ceiling levels of pre-tone context freezing. However, in Context C where the rats' freezing was almost absent, the difference between freezing to the tone CS and that to the context was significant [paired *t*-test, *t*_(7)_ = 3.294, *p* = 0.013], indicating that the CS-evoked freezing is a dominant response in CDFD.

### General locomotion and sensory gating are not disrupted by PL lesion

**Figure A1:**
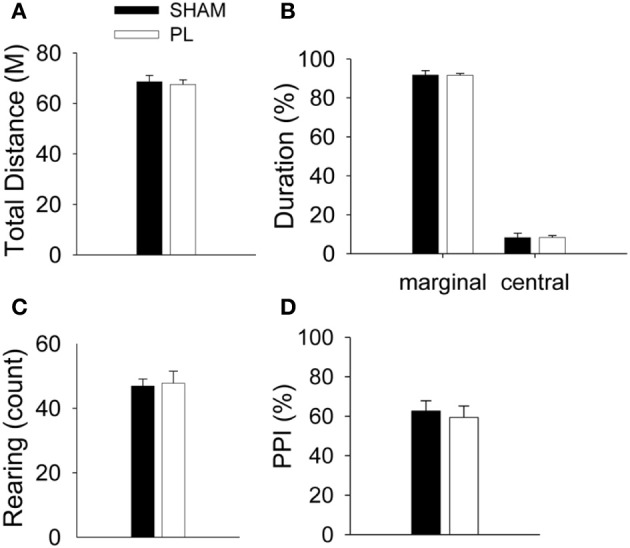
**Effects of PL lesions on general activity and sensory gating**. There were no differences between groups in total distance **(A)**, the ratio of the duration in marginal and central area **(B)**, and rearing **(C)**. In the PPI test, the ratio of inhibition was not different between groups **(D)**.

To test the effect of the PL lesion on ambulatory and emotional response, the open field test was conducted following completion of the CDFD task. There were no significant differences between the SHAM and PL lesion groups in total distance [*t*_(14)_ = 0.412, *p* > 0.6, Figure A1A, time spent in the marginal area [*t*_(14)_ = 0.362, *p* > 0.7, Figure A1B], and the frequency of rearing [*t*_(14)_ = 0.204, *p* > 0.8, Figure A1C]. These results indicate that the PL lesion did not impair the general activity level, emotional state, or exploratory behavior of the rats.

Damage to the prefrontal cortex often leads to alterations in the sensory gating (Robin and Holyoak, 1995; Christoff et al., 2001; Wright et al., 2009), which potentially compromises a rat's ability to discriminate context. To examine sensory gating in the PL lesion group (Koch and Schnitzler, 1997), the PPI test was conducted after the open field test. An independent *t*-test showed that the PL lesion group was no different from SHAM group in regards to the PPI [*t*_(14)_ = 0.412, *p* > 0.6, Figure A1D].

### PL lesion spares simple context discrimination

To examine whether the effect of the PL lesion on the CDFD was due to disruption in context discrimination, an additional experiment was conducted with a separate group of animals.

**Figure 3:**
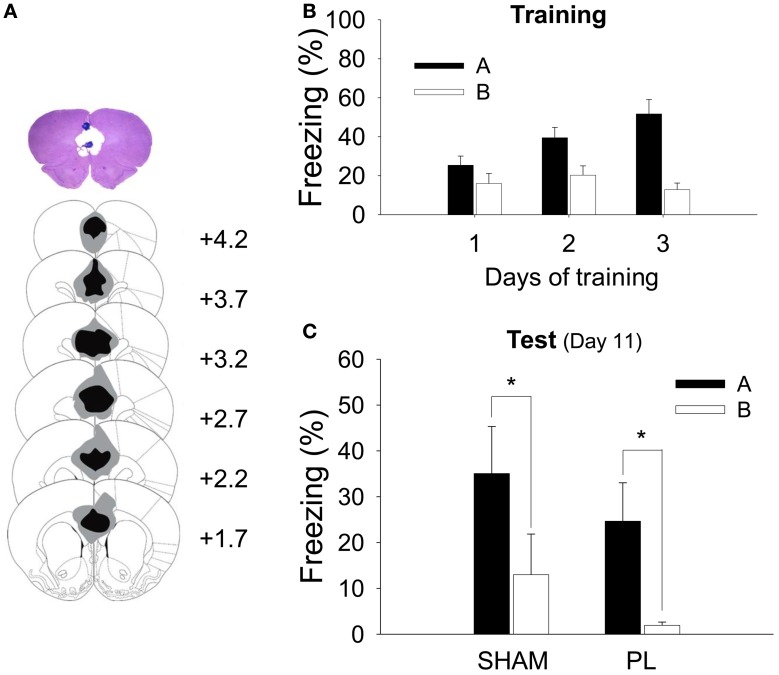
**Histological verification of the PL lesion and simple context discrimination. (A)** A high-resolution scan of a cresyl-violet-stained coronal section shows a representative PL lesion (top), and the reconstruction shows the extent of the electrolytic lesion (bottom). The gray shading depicts the largest and the black depicts the smallest lesion on the matching coronal sections (Paxinos and Watson, 1998). **(B)** During training, all animals gradually acquired fear responses to Context A but not to Context B. **(C)** After the surgery, both SHAM and PL lesion groups continued to show differential response to the two contexts as indicated by a higher level of freezing to Context A than that to Context B. ^*^denotes *p* < 0.05.

Figure 3A shows the extent of the electrolytic lesion in the PL lesion group (83.01 ± 5.49%, mean ± SD). The anterior portion of the Cg1 (36.15 ± 11.51%) and the dorsal IL (16.52 ± 12.35%) were partially damaged by a lesion. The extent of the PL lesion was similar to that in the CDFD experiment.

All rats received 2 sessions of training for 3 days, in which they were given 3 shocks in Context A and no shock in Context B. Because there was no difference between groups (SHAM and PL lesion) in their freezing during training [group effect, *F*_1, 14_ = 0.036, *p* > 0.8; day × group interaction, *F*_2, 28_ = 1.457, *p* > 0.2; context × group interaction, *F*_1, 14_ = 0.027, *p* > 0.8; day × context × group interaction, *F*_2, 28_ = 1.360, *p* > 0.2], the two groups were collapsed for the training data. Before surgery, all rats showed more freezing to Context A than to B (Figure 3B). A RM ANOVA revealed a significant difference in freezing developing over days [day, *F*_2, 30_ = 8.857, *p* < 0.001; context, *F*_1, 15_ = 19.444, *p* < 0.001; day × context, *F*_2, 30_ = 28.042, *p* < 0.0001]. A further analysis demonstrated that differential response was evident on training days 2 (*p* < 0.005) and 3 (*p* < 0.0001). Twenty-four hours later, half of the rats were given a PL lesion and the other half a sham lesion. Seven days later, they were tested in Context A and then B (or B then A). All animals froze more in Context A than in B (Figure 3C). There was a significant context effect [*F*_1, 14_ = 15.101, *p* < 0.002], but no group effect [*F*_1, 14_ = 1.223, *p* > 0.2] or interaction [*F*_1, 14_ = 0.003, *p* > 0.9]. There was a significant difference in freezing between Context A and B in both groups (*p*s < 0.05), confirming the ability to discriminate was intact in both groups (35.1 ± 10.25, SHAM, Context A; 13.0 ± 8.87, SHAM, Context B; 24.7 ± 8.4, PL, Context A; 1.9 ± 0.7, PL, Context B).

### Single-unit activity in the PL is context-specific

**Figure 4:**
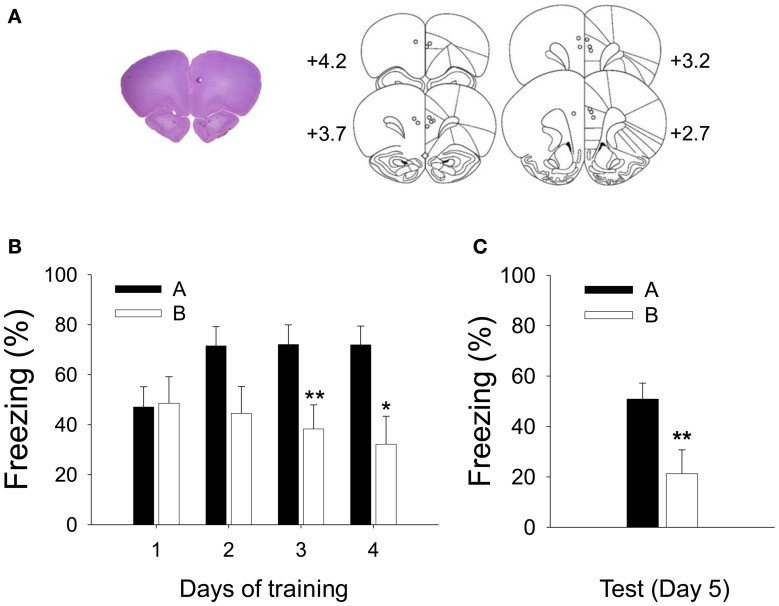
**Recorded locations and acquisition of CDFD. (A)** A high-resolution scan of a cresyl-violet-stained coronal section shows a representative electrode placement in the PL marked by a small lesion (left), and the reconstruction of electrode placements from all subjects (right) shows that the recording locations were confined within the PL. **(B)** Robust CDFD was developed following 4 days of training as all animals exhibited significantly greater freezing to the CS in Context A than in Context B on days 3 and 4. **(C)** On the test session, they also showed significantly more freezing to the CS in Context A than in Context B. ^*^ and ^**^denote *p* < 0.05 and *p* < 0.01, respectively.

To determine how PL neurons encode CS information in different contexts in CDFD, single-unit activities in the PL were recorded. Figure 4A shows a reconstruction of the recording electrodes and a representative brain section with tip locations.

The rats were trained with CDFD for 4 days. Freezing was analyzed by a RM ANOVA. A differential fear response developed over the training period as indicated by significant effect of context [*F*_1, 7_ = 7.099, *p* < 0.05] and day × context interaction [*F*_3, 21_ = 4.985, *p* < 0.01]. A *post-hoc* analysis revealed that the rats froze more to the CS in Context A than in B on training days 3 (*p* < 0.01) and 4 (*p* < 0.05) (Figure 4B). Twenty-four hours after the final training session, freezing to the CS was tested in Context A and then in B (or in B then in A, counterbalanced). The animals froze more to the CS in Context A than in B (Figure 4C) as evidenced by significant context effect [*F*_1, 7_ = 13.775, *p* < 0.01].

**Figure 5:**
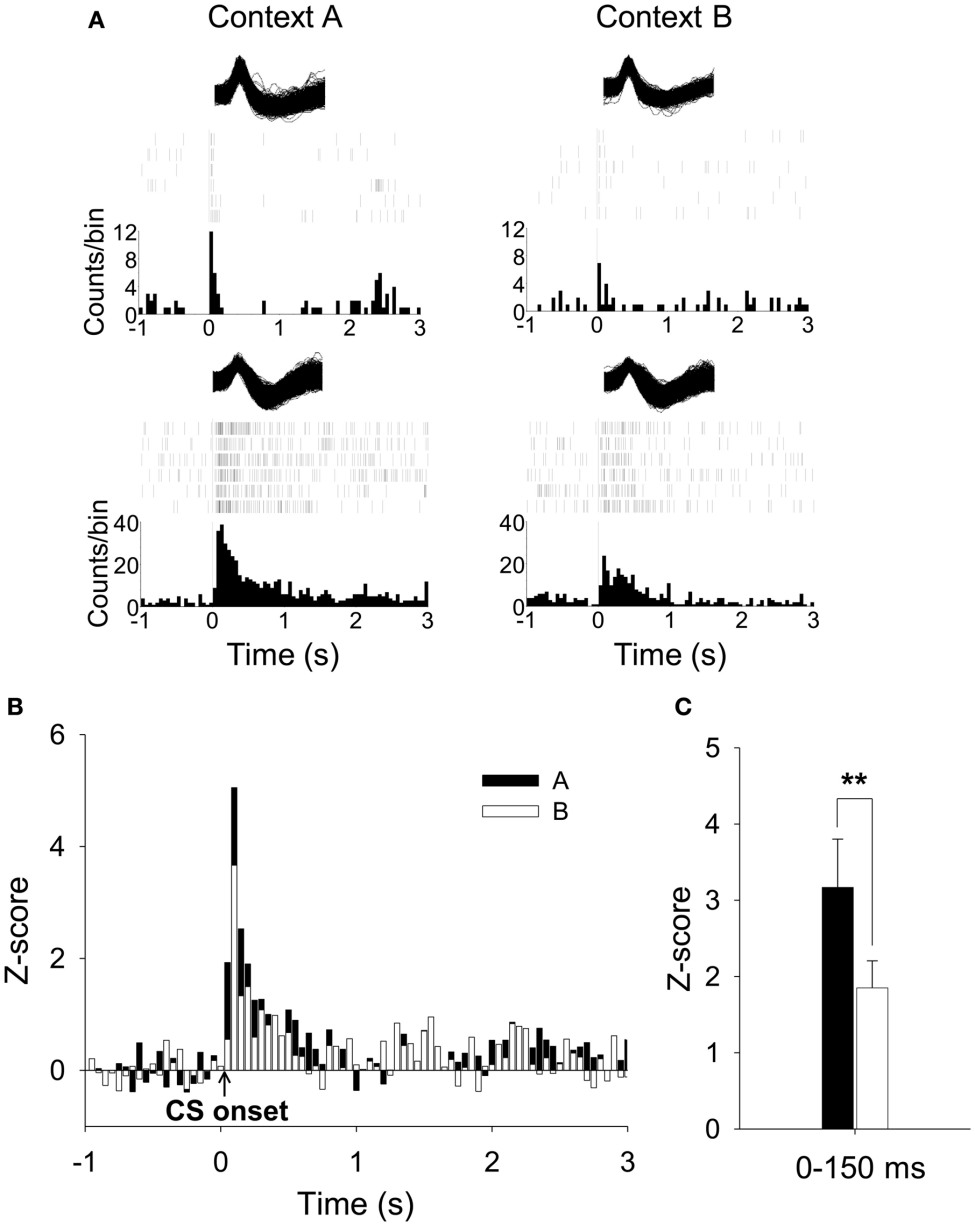
**Recorded activities of short-latency CS-responsive PL units during CDFD. (A)** Waveforms of two representative short-latency CS-responsive units, their raster plots, and the peristimulus time histograms (PSTHs) are presented. Both units showed increased firing rates to the CS in Context A than in Context B. **(B)** Overall activity of short-latency CS-responsive PL units during the CS presentation was greater in Context A than in Context B. **(C)** The mean firing rate during the initial 150-ms period after the CS onset was significantly higher in Context A than in Context B. ^**^denotes *p* < 0.01.

During the test session, a total of 72 units were isolated from 8 rats. Among them, 27 units (37.5%) were short-latency CS-responsive. Because context modulation of the CS was the primary interest of the current study, we mainly analyzed the CS-responsive units. To reveal any context-induced changes in spontaneous activity, firing rates during the 3-min baseline period were compared. The average firing rates varied (0.31–34.60 Hz) but did not differ significantly between the 2 contexts (paired *t*-test, *p* > 0.2; 6.09 Hz ± 1.42, for Context A; 7.56 Hz ± 1.81 for Context B). The spontaneous firing rate of PL units collected in the present study was comparable to that from previous studies (Baeg et al., 2001; Milad and Quirk, 2002). Figure 5A shows the representative waveforms, raster plots, and PSTH for the short-latency CS-responsive neurons. Figure 5B shows the average firing pattern of all short-latency CS-responsive units in Contexts A and B during the 1-s pre-CS and 3-s post-CS periods. PL neurons exhibited a higher rate of CS-evoked firing in Context A than in B, paralleling the differential freezing to the tone in the 2 contexts (Figure 4C). In particular, differential firing was more pronounced immediately after the CS onset. The firing rate of the short-latency CS-responsive units during the initial 150 ms of the tone presentation in Context A was significantly greater than that in B [paired *t*-test, *t*_(26)_ = 2.809, *P* < 0.01] (Figure 5C).

**Figure 6:**
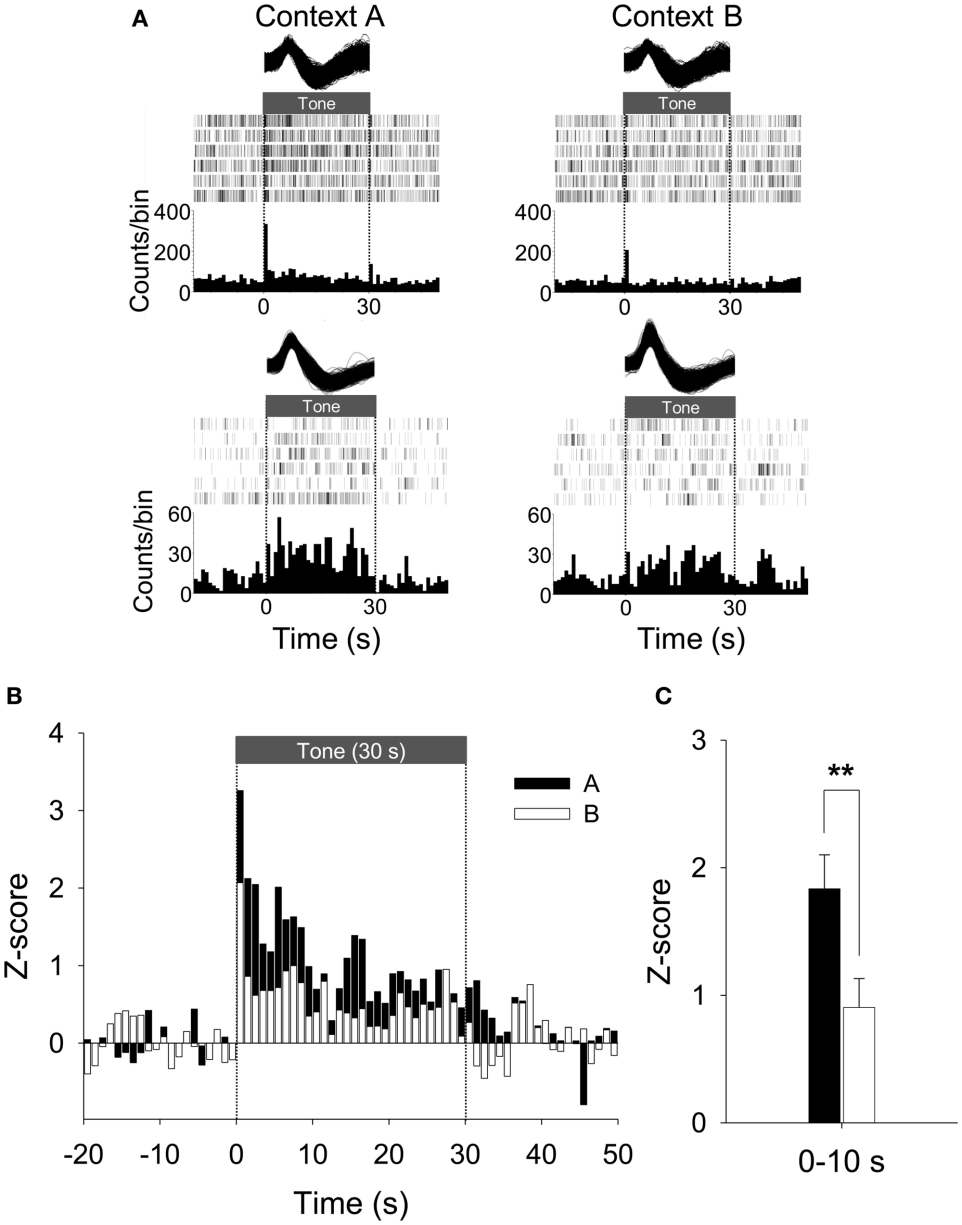
**Representative and average activity of PFUs during CDFD. (A)** Two representative waveforms of CS-responsive neurons and their raster plots and PSTHs are presented. Both units showed an increased firing rate to the CS in Context A than in Context B. Note that the first representative unit is the same as shown in Figure 5A. **(B)** The population firing pattern of all CS-responsive units in Context A and Context B are illustrated. The spike firing of PFUs to the CS was higher in Context A than in Context B. **(C)** The mean firing rate during the initial 10-s period after the CS onset was significantly higher in Context A than in Context B. ^**^denotes *p* < 0.01.

**Figure 7:**
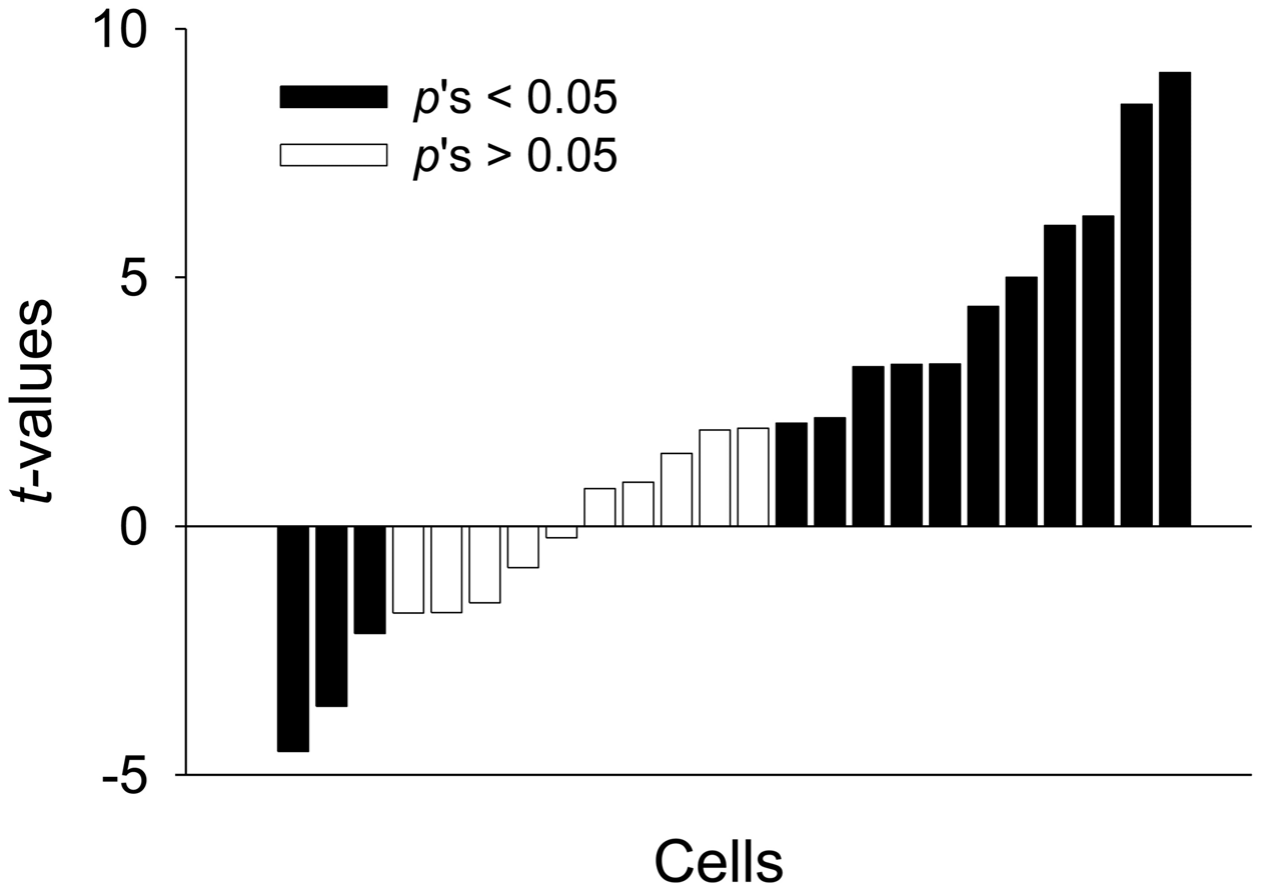
**Modulation of activity in PFUs**. The direction and amplitude of the modulation are expressed as paired *t*-values for each unit, which were computed from *Z*-score differences between Context A and B. The *t*-values were arranged in the order from the most negative to most positive values. A positive value indicates more activity in Context A. The dark bars represent statistically significant differences in firing rate between the two contexts and white bars represent non-significant differences.

To further explore whether a subset of neurons fired in a “persistent” manner, the unit data were binned into 1 s and normalized to the 20 baseline bins (20 s). We found 33% of PL units (24/72) were PFUs. Figure 6A shows the representative waveforms, raster plots, and PSTHs for the PFUs. Consistent with previous work (Burgos-Robles et al., 2009; Sotres-Bayon et al., 2012), the average firing rate of these PFUs during the entire CS presentation in Context A was significantly greater than that in B, as indicated by *Z*-scores [paired *t*-test, *t*_(23)_ = 2.37, *p* < 0.05, Figure 6B]. The differential firing was noticeable during the first 10 s [paired *t*-test, *t*_(23)_ = 2.96, *p* < 0.01, Figure 6C]. Among the 24 PFUs, only 11 units were also categorized as having short-latency response. Since there were 27 short-latency CS-responsive units, one possibility is that the remaining PFUs without the short-latency response (13 units) might have been triggered by the units showing short-latency responses and maintained their tonic activity by a recurrent network within itself or by recruiting additional areas. Also, to determine whether a given unit shows statistically significant modulation of firing rate in Context A in comparison to B, the PFU data were further analyzed by computing *t*-values based on paired comparisons between the matching bins. There were thirty 1-s bins for the duration of the tone CS and the *t*-values were calculated by summing the *Z-value* differences (Context A vs. B) for each bin. Among the units that have modulated their firing rates significantly during the presentation of the CS, 46% (11/24) showed significant positive *t*-values, whereas only 13 % (3/24) showed significant negative *t*-values. A chi-square test revealed that a higher percentage of tone-responsive neurons have positive *t*-values than negative *t-values* (χ^2^ = 6.454, *p* < 0.05, Figure 7). These results indicate that neurons in the PL fired more to the CS in Context A than in Context B.

Generally, fear responses decrease when the subjects are presented with repeated CS-alone trials, indicating within-session habituation which eventually contributes to extinction (Hefner et al., 2008). The within-session habituation is evident at the neuronal level, especially when massive extinction trials were used. It has been shown that the PL neurons exhibited decreased firing rate during the late (e.g., 19–20 trials) compared to early (e.g., 1–2 trials) extinction trials (Burgos-Robles et al., 2009; Sotres-Bayon et al., 2012). We also tested the possibility of within-session habituation of neuronal activity by dividing the 6 trials in each context into two 3-trial phases and calculating the *Z*-scores in each phase. We found no difference between the two phases. Specifically, the *Z*-scores significantly differed between the two contexts for both the short-latency response (mean *Z*-values during the 0–150 ms period; bin size: 50 ms) and sustained response (mean *Z*-values during the whole CS duration; bin size: 1 s) data [main effect of context; short-latency response: *F*_1, 21_ = 10.132, *p* < 0.01; sustained response: *F*_1, 23_ = 7.734, *p* < 0.05], but there was no phase effect [short-latency response: *F*_1, 21_ = 0.162, *p* > 0.6; sustained response: *F*_1, 23_ = 0.760, *p* > 0.3] or context × phase interaction [short-latency response: *F*_1, 21_ = 0.701, *p* > 0.4; sustained response: *F*_1, 23_ = 2.800, *p* > 0.1]. Note that 5 out of 27 units analyzed for the 50-ms bin data did not fire during the pre-CS baseline (1 s) throughout the initial or last 3 trials and were excluded from this analysis. These data exclude the possibility that a short-term habituation-like process across 6 trials during the recording session might have reduced the PL activities implicated in context-specific fear expression.

## Discussion

In this study, we demonstrated the regulatory role of the PL on the differential expression of learned fear by devising a task in which the context dictates CS–US contingency. In the CDFD task, a post-training PL lesion disrupted the differential expression of conditioned fear. However, the lesion effect on the CDFD task was not due to the inability to discriminate different contexts *per se*, since simple context discrimination was not impaired by the lesion. Moreover, CS-evoked activities in the PL neurons were selectively increased in the fearful context, paralleling the behavioral expression of the fear response. These results indicate that the PL plays a critical role in regulating a learned fear response, selectively enabling the expression of the fear response when the CS–US contingency is limited by contextual information.

**Figure A2:**
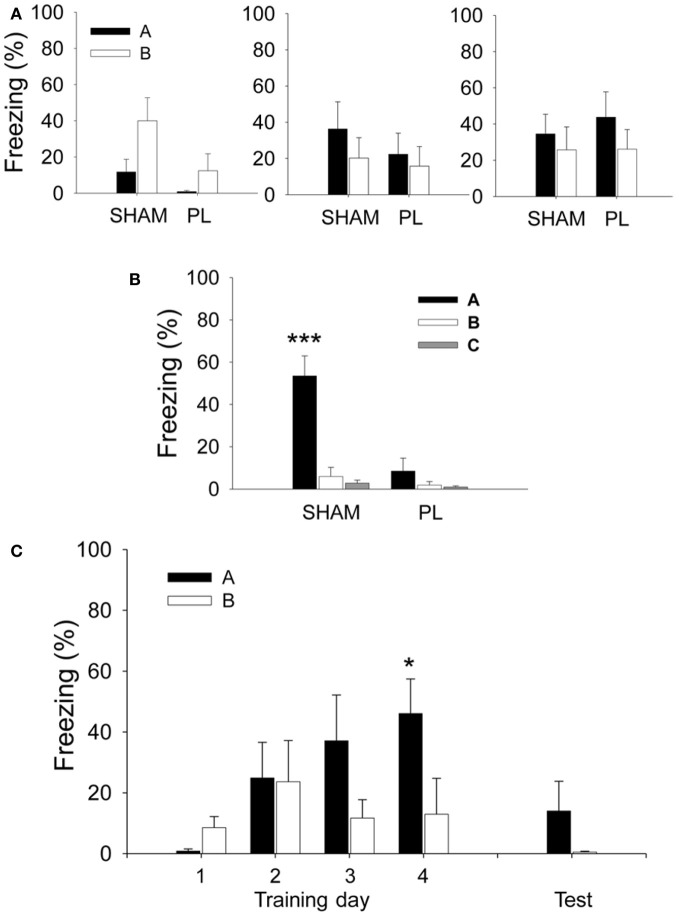
**Mean percentage of freezing during the pre-tone period before the presentation of the first CS**. (A,B) In the lesion experiment, both SHAM and PL lesion groups showed a similar level of pre-tone freezing across the 3 days of training **(A)**. However, on the test session **(B)**, the SHAM group developed a pre-tone response that discriminates Context A from other contexts, as opposed to the PL lesion group which continued to show non-differential responses in all three contexts. **(C)** In the recording experiment, the rats displayed similar levels of pre-tone freezing in Context A and B across the first three training sessions and testing, but not on training day 4 in which they showed differential responses. ^*^ and ^***^denote *p* < 0.05 and *p* < 0.001, respectively (RM ANOVA followed by Fisher's least significant difference test).

Considering that context dictates the shock contingency in CDFD, it is possible that direct association between the context and the US might have contributed to differential responses in the current study. No regulatory role for the context needs to be assumed if the rats chose the appropriate fear response based on contextual discrimination alone. However, the control rats froze significantly more to the tone in the novel context than in the safe context, indicating that context discrimination cannot sufficiently account for the differential responses. Evidently, the tone CS was a more salient stimulus than the contexts and directly controlled the rat's freezing. If the rats had learned to ignore the CS and chose their response solely based on context–US association, the level of freezing to the CS in the novel context should have been close to the baseline. Instead, an intermediate level of freezing to the CS, a level that was significantly higher than that in the safe context, was observed in the novel context. This suggests that the fear response triggered by the tone CS was “gated,” rather than directly produced by the context only (Figure 2C). Note, however, that the rats in the current CDFD task were not completely free from the direct context–US association as the rats showed some level of freezing to the context itself (Figure A2). Similarly, context freezing was frequently observed even during the pre-tone period of the test session when the rats were tested with ABA renewal design (Holt and Maren, 1999; Graham and Richardson, 2010).

Our lesion data present seemingly puzzling results regarding the levels of freezing to Context A vs. Context B: the differential response of PL-lesioned animals was indistinguishable from the control animals in the simple context discrimination task while the lesioned animals showed attenuated differential response across different contexts in CDFD as shown in Figure A2B. The latter result is also inconsistent with a previous study (Corcoran and Quirk, 2007). One possibility is that the context information is processed via different contingencies, i.e., more direct “context–US association” vs. “context–(CS–US) relation” (Bouton and Swartzentruber, 1986; Holland and Bouton, 1999). In addition to the direct context–US association, the rats also might have been subject to learning the regulatory nature of contextual information which dictates the CS–US in CDFD. It is possible, therefore, that the differential freezing between the two contexts observed in our study represented anticipatory responses to the CS in two different contexts. Since the mPFC is likely to engage in higher-order reasoning processes (Robin and Holyoak, 1995; Christoff et al., 2001), the PL might be recruited to process additional dimension of information posed by CDFD. Consistent with this view, microstimulation of PL elicited increased freezing when the stimulation was paired with the CS, but not without the CS (Vidal-Gonzalez et al., 2006).

Of notable interest was the failure of the rats to show a differential response to the CS in the PL lesion group was due to a reduction in freezing to the CS in the fearful context (Context A) as shown in Figure 2C. This indicates that the PL is involved in activating the fear circuit, consistent with several previous studies. For example, PL activation was necessary for the expression of the fear response (Corcoran and Quirk, 2007; Burgos-Robles et al., 2009), and inactivation of the PL reduced the level of fear response (Sierra-Mercado et al., 2006). However, the current result cannot be explained by a simple performance deficit or association failure because the PL lesion did not disrupt freezing in the simple context discrimination task (Figure 3C).

The low level of fear response in Context B developed gradually, resembling the course of extinction learning in which an initially high level of fear response declines over repeated presentation of the CS without the US. We observed that the level of freezing to the CS in both contexts was equally high at the beginning of training (Figure 2). These data suggest a common underlying mechanism between extinction and development of differential response in the current procedure. It has been generally accepted that extinction in Pavlovian conditioning is not an erasure of memory but rather a formation of new memory that alters the response to the original memory (Pavlov, 1927; Rescorla and Heth, 1975; Bouton and King, 1983; Baum, 1988). Note that the inhibition of freezing response in Context B in PL-lesioned rats could have resulted from the intact IL since the inhibitory process has been known to depend on the activity of the IL rather than the PL (Quirk et al., 2000; Milad and Quirk, 2002; Sotres-Bayon et al., 2006). Inhibition of fear response in the safe context in the PL lesion group, therefore, was preserved, perhaps due to intact IL. That being said, whether an antagonistic interaction between PL and IL plays a critical role in shaping and expressing fear response in various circumstances, however, awaits a further study (Sotres-Bayon and Quirk, 2010).

One might pose a possibility that the lesions extending to Cg or IL might have contributed to the deficits in CDFD performance. However, it is unlikely that the partial lesion in those areas would have attenuated the discriminatory response because the lesion effect observed in our study contracts what might be expected from Cg or IL lesion. For example, IL lesion or inactivation facilitated recovery of extinguished fear response (Quirk et al., 2000; Laurent and Westbrook, 2009), and extinction process was delayed in the Cg-lesioned rats (Morgan and LeDoux, 1995). Since most subjects in our lesion group showed decreased, rather than increased, freezing, it is reasonable to conclude that the additional lesions in Cg or IL did not contribute to the discrimination deficit.

Considering the fact that the current procedure could involve a significant extinction component which is known to be context-specific, another possibility is that the PL lesion impaired the context-specific inhibition on the fear response: i.e., over-generalization of extinction. The fact that the PL lesion lowered the level of freezing not only in Context A (excitatory context) but also in Context C (neutral context) without a significant change in freezing in Context B (inhibitory context) supports this interpretation. Normally, the inhibition of the conditioned response formed as a result of extinction training is vulnerable to context shifts (Bouton, 2004). The current results seem to suggest, however, that the PL lesion render the inhibitory process more robust and free from context requirement, without necessarily making the animal blind to contextual discrimination. While this is a viable possibility, it is not clear whether the PL significantly contributes to the extinction process. For example, no significant changes in the activation of the PL have been observed during simple fear extinction (Milad and Quirk, 2002; Chang et al., 2010). In addition, multiple studies suggest that the PL plays roles in expression of fear response (Vidal-Gonzalez et al., 2006; Corcoran and Quirk, 2007; Burgos-Robles et al., 2009) and in contextual control during operant conditioning (Marquis et al., 2007).

Interesting parallels should be noted between the current results and a study employing appetitive discrimination tasks. Haddon and Killcross (2006) trained rats in an auditory or visual discrimination task which required an appropriate response among competing responses, given the contextual cues. Using this task, they found that lesions in the mPFC altered the rats' context-appropriate response to the cues. In another study, pharmacological inactivation of the PL, but not the IL, prevented the rats from performing context-dependent discrimination (Marquis et al., 2007). The task requirement in these studies can be likened to the current CDFD task, in which the contextual information is necessary for solving the ambiguity of the CS. Thus, the PL seems to be involved in a common executive function in both negative and positive affective dimensions.

Since neuronal activity that discriminates different contexts and responds to auditory cues was evident, it is reasonable to assume that the PL receives contextual information as well as simple sensory signals to regulate the fear response. The differential firing of PL neurons observed in our study (Figures 5, 6) supports the possibility that the PL is provided with contextual information by the hippocampus and utilizes this information to amplify the neuronal response to the CS in the amygdala. Evidence implicating the hippocampus in forming a multimodal representation, including spatial layout of the environment, is abundant (O'Keefe and Dostrovsky, 1971; Wilson and McNaughton, 1993; Hollup et al., 2001; Poucet et al., 2004). Anatomically, the PL receives massive projections from the hippocampus, both directly (Jay et al., 1989; Conde et al., 1995) and indirectly (Thierry et al., 2000). Functionally, hippocampal inputs to the PL exhibit synaptic plasticity such as long-term potentiation- or long-term depression-like phenomena and depotentiation (Laroche et al., 1990; Burette et al., 1997; Takita et al., 1999). In fact, it has been proposed that spatial representation in the hippocampus could be fed into the PL to strengthen context-appropriate synaptic plasticity in an *N*-methyl-d-aspartic acid receptor-dependent manner (Jung et al., 1998). Alternatively, the cortico-hippocampal interaction might be reciprocal, as accumulating evidence suggests that PL neurons share location-specific firing patterns with the hippocampal place cells. In one study, Hok et al. (2005) showed that PL neurons exhibited place field firing that could encode goal-related location. Moreover, hippocampal representation of a specific spatial location was altered with lesions in the mPFC (Kyd and Bilkey, 2003).

Combined with the anatomical arrangement that the PL sends projections to the amygdala (Vertes, 2004; Likhtik et al., 2005), the cortico-hippocampo-amygdala circuit might account for contextual regulation of CS-elicited fear response. Apparently, context-dependent fear to discrete stimuli is expressed via amygdala neurons (Hobin et al., 2003). This context-specific activation of the amygdala is disrupted by temporal inactivation of the hippocampus (Maren and Hobin, 2007), presumably due to an inability to represent spatial context (O'Keefe and Dostrovsky, 1971; Phillips and LeDoux, 1992). Moreover, recent work demonstrated that inactivation of the ventral hippocampus reinstated extinguished fear responses to the CS as well as PL activity, perhaps via interneurons adjacent to pyramidal neurons, indicating that the ventral hippocampus contributes to reducing fear and fear-related PL activation (Sotres-Bayon et al., 2012). In comparison, our data provide critical evidence that the PL is necessary for generating context-appropriate fear response, rather than a passive read-out of the contextual information, by showing that PL lesions disrupted CDFD performance (Figure 2C). In addition, PL neurons differentially fired to the same CS in different contexts, indicating that the tone-associated activities are involved in active selection and execution of the appropriate fear response (Figures 5, 6). Together, the current results suggest that the PL might mediate contextual modulation of the fear response by controlling amygdala activity.

Other studies have emphasized the hippocampo-amygdala circuit in direct association of the context and shock (Maren and Fanselow, 1995, 1996; LeDoux, 2000) as well as in context-appropriate fear expression (Maren and Hobin, 2007). Since the PL receives auditory information from the auditory cortex (Conde et al., 1995) and fear information from the amygdala (Cassell et al., 1989; Sesack et al., 1989; Conde et al., 1995; McDonald et al., 1996), it is also possible that the PL might convey the combined information to the hippocampo-amygdala network when context-related fear processing becomes complicated. In support of this view, a recent study found that disruptions of the interconnections between the PL and the hippocampus or the basal nucleus of the amygdala impaired fear renewal in rats (Orsini et al., 2011). The PL is likely to be engaged in regulating a learned fear response, selectively enabling the expression of the fear response when the CS–US contingency is limited by contextual information. Differential firing of PL neurons also supports the regulatory account in fear expression (Figures 5, 6). However, whether and how the PL modulates fear-related synaptic plasticity occurring between the hippocampus and amygdala would require a separate experiment.

In summary, the PL might be crucial for fear expression when fear responses should be regulated by contexts. Without the PL, animals were not able to show the pertinent fear response to the CS in the appropriate fearful context after the acquisition of the discriminatory behavior to the discrete fearful stimulus. Considering the strong anatomical and functional connections between the PL and the hippocampus, further studies are needed to identify the exact role of the PL-hippocampus network in context-appropriate fear process. Although animals would choose to promptly express fear when confronted with a threat for survival, optimal fear responses are gradually developed by repeated exposure to the same or similar circumstances to modify inadequate behaviors and successfully adapt to the environment. Emotional disorders, such as posttraumatic stress disorder, depression, and addiction, can be caused by deficits in the cognitive control of emotion (optimization processes), which recruits higher executive function of cortical areas. Therefore, lowering the activity of a human brain area that corresponds to the PL might effectively alleviate the symptoms of fear-related disorders.

### Conflict of interest statement

The authors declare that the research was conducted in the absence of any commercial or financial relationships that could be construed as a potential conflict of interest.
